# Primary care priorities in addressing health equity: summary of the WONCA 2013 health equity workshop

**DOI:** 10.1186/s12939-014-0104-4

**Published:** 2014-11-07

**Authors:** Efrat Shadmi, William CW Wong, Karen Kinder, Iona Heath, Michael Kidd

**Affiliations:** Faculty of Social Welfare and Health Sciences, University of Haifa, Haifa, Israel; Department of Family Medicine and Primary Care, The University of Hong Kong, Pokfulam, Hong Kong; ACG International, Johns Hopkins University, Baltimore, MD 21218 USA; Royal College of General Practitioners, London, UK; WONCA, Flinders Medical School, Flinders Dr, Bedford Park, SA 5042 Australia

**Keywords:** Health equity, Disparities, Primary care

## Abstract

**Background:**

Research consistently shows that gaps in health and health care persist, and are even widening. While the strength of a country’s primary health care system and its primary care attributes significantly improves populations’ health and reduces inequity (differences in health and health care that are unfair and unjust), many areas, such as inequity reduction through the provision of health promotion and preventive services, are not explicitly addressed by general practice. Substantiating the role of primary care in reducing inequity as well as establishing educational training programs geared towards health inequity reduction and improvement of the health and health care of underserved populations are needed.

**Methods:**

This paper summarizes the work performed at the World WONCA (World Organization of National Colleges and Academies of Family Medicine) 2013 Meetings’ Health Equity Workshop which aimed to explore how a better understanding of health inequities could enable primary care providers (PCPs)/general practitioners (GPs) to adopt strategies that could improve health outcomes through the delivery of primary health care. It explored the development of a health equity curriculum and opened a discussion on the future and potential impact of health equity training among GPs.

**Results:**

A survey completed by workshop participants on the current and expected levels of primary care participation in various inequity reduction activities showed that promoting access (availability and coverage) to primary care services was the most important priority. Assessment of the gaps between current and preferred priorities showed that to bridge expectations and actual performance, the following should be the focus of governments and health care systems: forming cross-national collaborations; incorporating health equity and cultural competency training in medical education; and, engaging in initiation of advocacy programs that involve major stakeholders in equity promotion policy making as well as promoting research on health equity.

**Conclusions:**

This workshop formed the basis for the establishment of WONCA’s Health Equity Special Interest Group, set up in early 2014, aiming to bring the essential experience, skills and perspective of interested GPs around the world to address differences in health that are unfair, unjust, unnecessary but avoidable.

## Background

“Inequity is built into health systems- especially health systems that are based on a view of health needs disease by disease. Therefore, the benefits of primary care, which is person- and population- rather than disease-focused, are underappreciated. Data provide evidence not only of its benefit to populations but also of its preferential benefit to the socially disadvantaged” (Starfield, [1]).

Health inequities occur within socioeconomic classes, [2–5] and span across a wide range of socio-cultural characteristics [2,6]. Health inequity refers to differences in health that are not only unnecessary and avoidable, but are also unfair and unjust [7]. Research consistently shows that gaps in health and health care persist, and are even widening [8,9]. Such evidence is triggering renewed interest by politicians and policymakers in development and implementation of interventions to reduce inequity; however, there is little knowledge on how to effectively achieve inequity reductions [10].

Several reasons have been provided for this intolerable gap between the realization of the expansion of inequity in health and the paucity of evidence on how to reduce it, including limitations in the design and implementation of interventions, and the complexity of addressing the social determinants of health [8]. A special concern is the understanding that health care plays a relatively minor role in explaining health inequities, and that the relative contribution of health services to tackling inequity in health, as compared to the role of the wider social determinants of health, is probably small [10].

Such conclusions, however, ignore evidence that has repeatedly shown that the strength of a country’s primary health care system and its primary care attributes significantly improves populations’ health and reduces inequity [11–16]. Starfield *et al.* identified the primary care attributes that contribute to population health, including first contact access, greater focus on prevention, provision of person-focused comprehensive care, with greater continuity and coordination [14]. Such attributes are of special importance to inequity reduction as the socially disadvantaged have a greater likelihood of occurrence, severity, and adverse effects in multiple illnesses for which a comprehensive, coordinated, person-focused (rather than a specialty driven disease-focused) view of morbidity can be more effective [1].

Recent evidence supports the above conclusions showing that primary care can reduce inequity in developed as well as in low- and middle-income countries. For example, in an observational study of all 152 English primary care trusts, Levene and colleagues [17] showed that variations in primary healthcare services could predict variations in mortality at the population level, after adjusting for population characteristics. Specifically, that study showed that being able to see a preferred doctor (linked to both access and continuity) was related to differences in cancer and chronic obstructive pulmonary disease mortality and that the barriers to forming sustained partnerships with primary care providers (PCP) were greater in deprived populations. A recent review that assessed the contribution of large primary care initiatives to a broad range of health system goals in low- and middle-income countries concluded that primary care-focused health initiatives had improved access to health care, including among the poor, at reasonably low cost and primary care programs had reduced child mortality and, in some cases, wealth-based inequity in mortality [18].

A suggested framework through which primary care interventions can work to reduce inequity is quality improvement (QI). QI has been described as effective in reducing inequity through designated teams and established goals and metrics, with leadership support and cultivation of local partnerships [19]. An example of how primary care QI can reduce inequity was provided by Balicer and colleagues who described how a primary care based inequity reduction strategy had improved quality and reduced inequity in seven health domains (diabetes, hypertension and lipid control, anaemia prevention in infants, performance of influenza vaccinations, and screening tests for breast and colorectal cancer) [20].

The role and achievements of primary care in reducing inequity uniquely position family practitioners as important advocators for expansion of primary care services for marginalized groups. One of many examples comes from Thailand, where largely as a result of the advocacy of the Rural Doctors Society, insurance for medical services was progressively expanded to cover the entire population of Thailand by the early 2000s. During this period, under-5 mortality was lowered by a much greater percentage in more deprived populations than in less deprived ones and both relative and absolute differences in under-5 mortality were reduced [21].

Despite this body of evidence on the relationship between the supply and attributes of primary care and reduced inequity, many areas such as the provision of health promotion and preventive services, are not explicitly addressed by general practice [22]. Substantiating the role of primary care in reducing inequity as well as establishing educational training programs geared towards health inequity reduction and improvement of the health and health care of underserved populations are needed [23].

### How could primary care contribute to this movement?

As a foundation step for the establishment of WONCA’s Health Equity Special Interest Group, The World WONCA (Global Organization of Family Doctors) held a workshop on health equity during its triennial meeting in Prague (26–30 June 2013). The aims of the workshop were to explore how a better understanding of health inequities could enable PCPs/general practitioners (GPs) to adopt strategies that could improve health outcomes through the delivery of primary health care.

The workshop addressed two orientations that provide the framework by which people identify issues as moral issues, namely an orientation of justice and orientation of care [24]. A justice orientation is concerned with issues of fairness, individual rights, and adherence to standards and principles. From this framework, morality requires following the universal ethical principles of justice, autonomy, reciprocity, equality, and respect for all human beings. A care orientation framework is concerned with the complexities of sustained attachments, compassion, forgiveness, and close personal relationships. From a care perspective, morality requires not hurting others, condemning all violence and exploitation, and nurturing relationships and connections between persons. These two frameworks guided the workshop’s discussions and were addressed in the opening presentations on Health and Justice (IH), pointing to the framing of inequity calling for GP’s advocacy and attention, [25] and on current initiatives and best practices in primary care (ES), suggesting the ways the structure and processes of care can alleviate the effects of inequity [26].

Specifically, the workshop also explored the development of a health equity curriculum and opened a discussion on the future and potential impact of health equity training among GPs. This report summarizes the results of structured group discussions held at the workshop and a survey on the current and expected levels of primary care participation in various inequity reduction activities in each respondent’s country.

## Methods

### Workshop program and participants

The workshop was led by the authors of this report, who served as moderators (WW and KK), provided the overview (MK) and presented the scope of health equity, challenges and problems pushing the agenda forward (IH) and a brief overview of current initiatives and best practices dealing with health equity in family medicine/ primary care setting (ES). It was attended by practicing primary care physicians, general practitioners and residents; a total of 120 delegates from across the globe, including low, middle and high-income countries from Europe, the Middle East, Asia, Africa, North and South America and Australia. Following the presentations, workshop attendees were asked to participate in small group discussions. In addition, the respondents were asked to rate on thirteen possible inequity reduction activities, the level in which the activity was currently part of their countries’ key priority issues and to which they believed these activities should be part of their countries’ key priority issues on a 1–5 Likert scale in an anonymous survey. Responses were summarized and the differences between actual and preferred level of involvement were calculated for each activity.

## Results

In the small group discussions, participants recognized how uneven distribution of social determinants of health could have affected poor health outcomes such as life expectancies and risk behaviours, and how health systems that operated in different countries could have systematically affected people’s affordability as well as access to healthcare services and fundamental rights to good health. Participants identified health workforce shortage, lack of communications between primary & secondary care, low political incentive & priority for marginalized populations as well as low health literacy & expectation of the patients as contributions in meeting the health equity agenda. They believed training on how to navigate the healthcare system and training should be provided to leaders of vulnerable groups since community awareness should be provided to the public as well as the patients. Furthermore, they believed that training in inequity should be provided to medical students as well as GPs on how to improve health equity through primary care. (A full report can be obtained: http://www.globalfamilydoctor.com/News/AddressingHealthEquity.aspx).

Table 1 reports the results of the survey on actual and preferred activities performed in efforts to reduce inequity. Overall, participants rated the degree to which their country currently utilizes the various mechanisms to reduce health inequity as moderate (mean: 2.85, SD: 1.12) with ratings ranging between 2.12 to 3.63. The results indicated that the types of mechanisms most commonly utilized included: promoting access to primary care services (mean: 3.63, standard deviation (SD): 1.24); initiation of public health programs to promote health equity (mean: 3.17, SD: 1.01); and, promoting access to care by increasing coverage of services (mean: 3.12, SD: 1.36). The activities least practiced were: engagement in cross-national collaborations to promote health equity (mean: 2.12, SD: 0.99); promoting research on health equity (mean: 2.40, SD: 1.04); and, reforming medical education to incorporate health equity and cultural competency training (mean: 2.44, SD: 0.96).

**Table 1:** **Activities aimed at reducing inequity in health**

	**The level to which this activity is currently part of respondent’s countries’ key priority issues (1–5)** ^*^ **, M ± SD**	**The level to which this activity SHOULD be part of respondent’s countries’ key priority issues (1–5)** ^**^ **, M ± SD**
1. Initiation of **advocacy programs** to involve major stakeholders in equity promotion policy making	2.71 ± 1.08	4.35 ± 0.75
2. Reform of **medical education** to incorporate health equity and cultural competency training	2.44 ± 0.96	4.19 ± 0.83
3. **Promote research** on health equity	2.40 ± 1.04	3.96 ± 0.85
4. Initiate **public health programs** to promote health equity	3.17 ± 1.01	4.22 ± 0.89
5. Initiate **primary care programs** to promote health equity	2.96 ± 1.04	4.42 ± 0.76
6. Promote **access** to care – availability of primary care services	3.63 ± 1.24	4.68 ± 0.63
7. Promote **access** to care- increased coverage of services / health insurance	3.12 ± 1.36	4.20 ± 1.12
8. Promote **access** to care- point-of- service free care	3.04 ± 1.06	4.08 ± 1.08
9. Promote the **collection** of socio-demographic data on patients in a routine and standardized way	3.07 ± 1.46	3.82 ± 1.31
10. Write **guidelines** for physicians for health equity promotion	2.70 ± 1.23	3.74 ± 1.13
11. Promote the development and implementation of tools to **measure and monitor** inequity in health	2.73 ± 1.12	4.11 ± 0.70
12. Promote **diversity** in medical workforce	2.96 ± 1.19	4.04 ± 0.84
13. Engage in **cross-national collaborations** to promote health equity	2.12 ± 0.99	4.08 ± 0.91

*1 = Currently not a priority; 5 = Currently a top priority.

**1 = SHOULD not be a priority; 5 = SHOULD be a top priority.

On average, participants rated the degree to which they believed the items representing priority areas which their governments and regional authorities should be engaged in as “high” (average: 3.85, SD: 0.88). The types of mechanisms most commonly viewed as practices that should be adopted were: promoting the availability of primary care services (mean: 4.68, SD: 0.63); and, initiation of primary care programs to promote health equity (mean: 4.42, SD: 0.76). The activities that are least viewed as those that should be promoted were: writing health equity promotion guidelines for physicians (mean: 3.74, SD: 1.13); and, collection of socio-demographic data on patients in a routine and standardized way (mean: 3.82, SD: 1.31).

**Figure 1:**
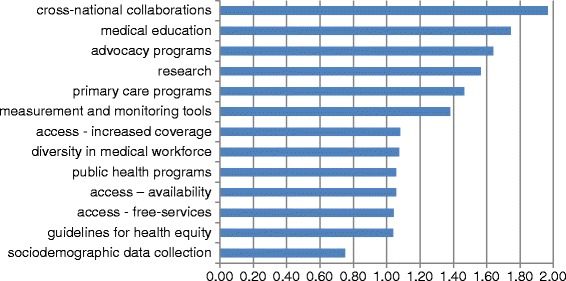
**Difference between actual performance and preferred inequity reduction activities.**

For each item there was an average of 1-point difference between rating of current and desired level of activity, indicating that respondents felt that much more should be done in each area currently underway. Differences ranged from 1.96; 1.75 and 1.64 for “engaging in cross-national collaborations to promote health equity”, “reform of medical education to incorporate health equity and cultural competency training” and “Initiation of advocacy programs to involve major stakeholders in equity promotion policy making”, to a 0.75 point difference for “collection of socio-demographic data on patients in a routine and standardized” respectively (see Figure 1).

## Discussion

Despite evidence of the contribution of the core attributes of primary care to populations’ health and reduction of inequity, inconsistencies are found in implementation of primary care features in different countries, with greater emphasis on the provision of easily accessible primary care and less investment in promoting programs to improve continuity or coordination [27].

Health equity workshop participants expressed similar assessments regarding their governments’ and health authorities’ priorities, indicating that promoting access (availability and coverage) of primary care services was more often performed than initiating tailored primary care interventions (mean score: 3.04-3.63, compared to 2.96, respectively).

Activities that have been previously cited as important for promoting equity in health – i.e., reform in medical education, promoting research, and fostering cross-national collaborations, were identified by survey respondents as low current priorities in their own countries. It is noteworthy that survey respondents identified “promoting access to primary care services” as the most important priority.

Furler and colleagues’ [28] used Gruen’s model, [29] to analyse physician responsibilities in defining how professional medical colleges and associations should lead the profession in responding to socioeconomic health inequalities. Their study showed that even areas that are defined by Gruen as professional obligation, such as reducing financial barriers to improve access to care, were contested and that some of the areas defined by Gruen as professional aspirations, such as advocacy, were viewed as integral roles of the profession. While we did not directly assess Gruen’s model, our results show that activities that can be defined as professional obligations, such as promotion of access or initiation of equity promoting programs, were ranked as more highly performed than activities such as advocacy or engagement in cross-national collaborations, that are defined by Gruen as professional aspirations. Interestingly, activities related to improved access and program initiation, as well as advocacy, were ranked as high priorities that health care systems and governments should engage in.

Following the prioritization outlined above there is a need to further examine how such obligations can be directly carried out by primary care professionals and not merely remain as aspirations to be addressed by governments. To help realize more practical goals and bridge between aspirations and obligations, it has been suggested to form a group within WONCA, [28] which can guide the further delineation of the ways in which access, intervention programs and education can provide a care orientation framework for equity promotion.

There are of course, limitations to our findings as the information reported here are not of a representative sample of countries, regions or professionals. Nonetheless, the reported priorities indicated here are viewed as areas of needed improvement also by other researchers and policy makers.

## Conclusions

Assessment of the gaps between current and preferred priorities indicated by the participants of the WONCA Health Equity workshop showed that to bridge expectations and actual performance, the following should be at the focus of governments and health care systems: forming cross-national collaborations; incorporating health equity and cultural competency training in medical education; and, engaging in initiation of advocacy programs that involve major stakeholders in equity promotion policy making as well as promoting research on health equity.

The World WONCA Health Equity Special Interest Group (SIG) was set up in early 2014 bringing the essential experience, skills and perspective of interested GPs around the world to address the differences in health that are unfair, unjust, unnecessary but avoidable. This group intends to use WONCA as a platform for exchange of ideas, advice, support and advocate for better equity in health. For more information on the Health Equity SIG: http://www.globalfamilydoctor.com/groups/SpecialInterestGroups/HealthEquity.aspx.
